# Prenatal and early life maternal substance use prevalence among Australian children born 2007-2018: a data linkage study using health, death, and child protection data

**DOI:** 10.23889/ijpds.v9i5.1680

**Published:** 2024-09-18

**Authors:** Madeleine Powell, Rhiannon Pilkington, Tasnia Ahmed, Mark Hanly, BJ Newton, John Lynch, Timothy Dobbins, Jessica Stewart, Michelle Cretikos, Alys Havard, Falster Kathleen

**Affiliations:** 1 School of Population Health, University of New South Wales; 2 National Drug and Alcohol Research Centre, University of New South Wales; 3 School of Public Health, University of Adelaide; 4 Centre for Big Data Research in Health, University of New South Wales; 5 Social Policy Research Centre, University of New South Wales; 6 Population Health Sciences, University of Bristol; 7 Family and Community Services Insights, Analysis & Research, Department of Communities and Justice, New South Wales Government; 8 Centre for Epidemiology and Evidence, New South Wales Ministry of Health

## Objective

Quantify the scale and type of maternal substance use from conception to the child’s second birthday (First 1000 days) to inform screening and support services that may reduce associated harm or risk for children.

## Approach

We used mother and child records from whole-population health, death, and child protection datasets to ascertain maternal substance use during the First 1000 days for children born in NSW, Australia, from 2008-2017. The primary outcome - maternal substance use - included use of illicit substances, alcohol, opioid-agonist treatment, organic compounds, solvents, and misuse of prescription medicines. ICD-10 and SNOMED-CT diagnosis codes were used.

## Results

The birth cohort included 970,470 children and 625,856 mothers. 32,000 children (3.4%) had a record of maternal substance use during the First 1000 days of life, including 13,647 (1.4%) with alcohol and 23,485 (2.4%) other drug use. Ascertainment was highest from child protection records (26,045 children), followed by mother’s (12,956 children) then children’s hospital records (3,826 children). 18,672 (1.9%) children had a record of carer substance use only in child protection records. Combining data increased the prevalence estimates; adding child protection records increased the prevalence estimate to 3.4%, compared with 1.2% in health and death records alone.

## Conclusion

More than 3 in every 100 Australian children had a record of maternal substance use in administrative data during the First 1000 days of life in this decade-long study. In addition to health and death data, child protection data offers public health insights into the scale of maternal substance use among whole-population cohorts of children.

